# Theoretical Study of Structural and Electronic Trends of the Sulfonylurea Herbicides Family

**DOI:** 10.1021/acs.jpca.4c03259

**Published:** 2024-07-16

**Authors:** Antonio Pulgar, Mónica Valentín, Clemens Rauer, Paula Pla, José-Luis Alonso-Prados, Pilar Sandin-España, Al Mokhtar Lamsabhi, Manuel Alcamí

**Affiliations:** † Departamento de Química, Facultad de Ciencias, Módulo 13, Universidad Autónoma de Madrid, 28049 Madrid, Spain; ‡ Plant Protection Products Unit/Plant Protection Department, National Institute for Agricultural and Food Research and Technology INIA-CSIC, Ctra. La Coruña, Km. 7.5, 28040 Madrid, Spain; § Institute for Advanced Research in Chemical Sciences (IAdChem), Universidad Autónoma de Madrid, 28049 Madrid, Spain; ∥ Instituto Madrileño de Estudios Avanzados en Nanociencias (IMDEA-Nanociencia), 28049 Madrid, Spain

## Abstract

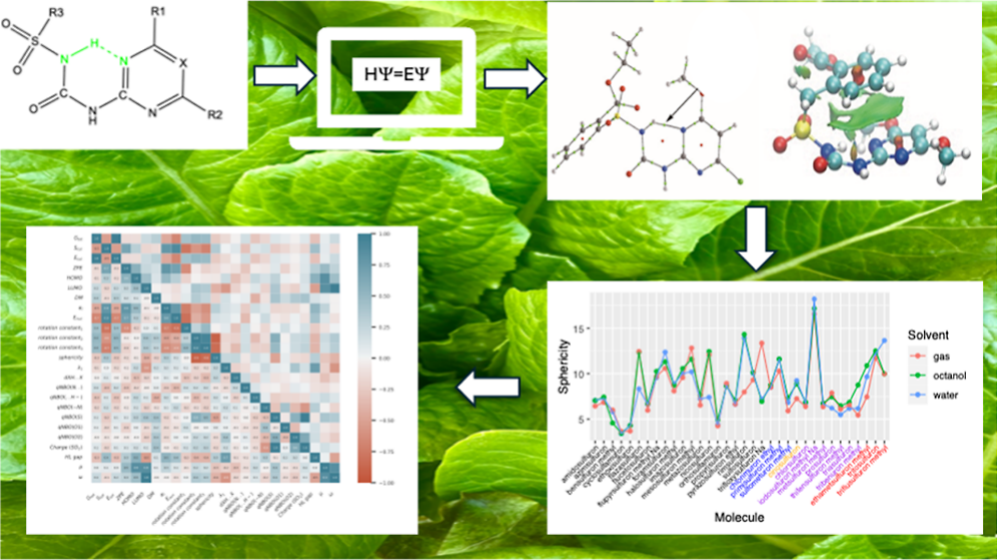

The sulfonylurea herbicide family has been extensively studied using computational techniques. The most stable conformer structures of the 34 molecules analyzed in gaseous, aqueous, and octanol phases have been determined. The study employed CREST conformational search methods along with the CENSO script to explore all possible conformational structures. Additional evaluations conducted at the B3LYP-D3/6-311+G(d,p) level have enabled the identification of intramolecular stability patterns across the various compounds. It has been discovered that stability is primarily determined by two factors: intramolecular hydrogen bonding involving an NH group adjacent to the sulfonyl group with either N donors or the nearby carbonyl group and potential π–π interactions between the aromatic rings of the molecules. These have been characterized through QTAIM and NCI population analyses. Furthermore, with the goal of developing predictive models for the physicochemical properties of pesticides that include the sulfonylurea family, a statistical analysis among the different properties of the studied molecules has been conducted. Significant correlations have been found between various properties, predicting a promising future for the prediction of characteristics that could assist laboratories in selecting among different pesticides.

## Introduction

Sulfonylurea herbicides play a pivotal role in weed and grass control across diverse crops such as wheat, rice, maize, barley, potatoes, soybeans, and blueberries. Their mode of action is rooted in their ability to inhibit acetolactate synthase. This enzyme is crucial for synthesizing branched-chain amino acids in plants.^1−3^ Their prominence as a group of herbicides can be attributed to several factors: (i) high efficacy; (ii) minimal mammalian toxicity; (iii) broad-spectrum application; and (iv) high selectivity that permits low application dosages. However, there’s an environmental concern. Due to characteristics like low volatility, significant water solubility, and potent leaching capability, sulfonylurea herbicides can migrate into aquatic ecosystems, posing water pollution risks.^4−7^ Nowadays, authorities at both national and international levels, as well as environmental agencies responsible for the authorization for commercialization of pesticides, have implemented stringent regulations to protect public health and the environment. Meeting this regulation involves performing risk assessments of the use of the pesticide and therefore implies a deeper knowledge of the fate and behavior of these compounds in the environment. In this sense, prediction tools can be highly useful in generating the information that must be included in the models and scenarios used to perform the risk assessment. A primary degradation mechanism of sulfonylureas herbicides is hydrolysis, especially under acidic conditions. This reaction’s rate is influenced by pH, with some studies also examining hydrolysis in various environments.^1,8^ While photolytic and photocatalytic are not the main degradation routes in the environment, these processes have been thoroughly researched.^9−12^

**Scheme 1:**
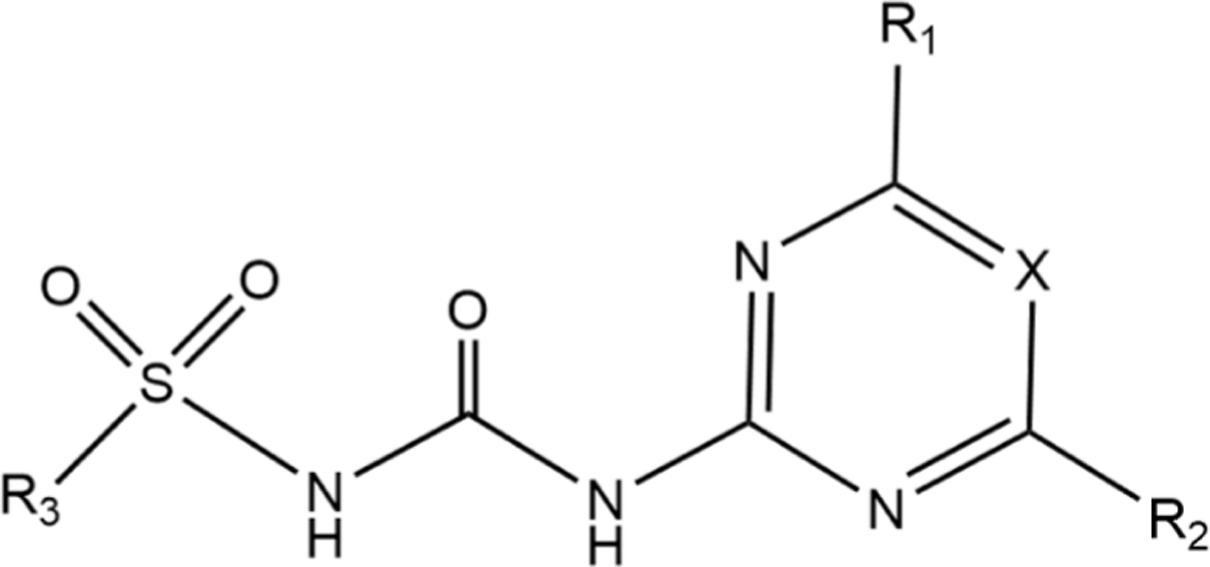
Structure of Sulfonylurea Herbicides

Structurally, sulfonylureas consist of a sulfonylurea bridge linking two distinct substituents (see Scheme 1). On the one end, there’s a heterocycle, either a diazine (pyrimidine) or triazine (1,3,5-triazine) derivative. The opposite end (R3) typically features an aromatic substituent.^1,13^

An intriguing aspect of sulfonylureas is their conformational flexibility. The physicochemical properties of these molecules are dependent on their substituents (R1, R2, and R3), as well as their spatial arrangement and the surrounding solvent. These factors may also have an impact on their environmental and toxicological properties. Moreover, the structural design of sulfonylureas allows them to exist in various tautomeric forms, including keto–enolic and imine–enamine tautomerism. A comprehensive understanding of these herbicides requires a holistic view of all potential conformations.

In the present study, we delve into a theoretical exploration of the entire sulfonylurea herbicides family, which according to Herbicides Resistance Action Committee (HRAC, https://hracglobal.com) encompasses 34 distinct compounds. Our analysis spans the gamut of various tautomers and conformers, meticulously evaluating each for stability and electronic characteristics. Beyond this, we have dedicated special attention to discerning structural nuances within these compounds. By doing so, we aim to elucidate how these structural variations intertwine with and influence their electronic properties. One objective of our study is to explore which of the electronic properties obtained in the calculations can be more suitable to be used as input parameters for QSAR models. In this respect, a deeper understanding of the structure of the compounds and how the structure influences the electronic properties may give also insight on how they can affect the QSAR predictions and will set the stage for advances in the prediction of their environmental and toxicological properties. QSAR models generally use experimental data but can also benefit from prediction obtained by quantum chemistry calculations.^14^

**Figure 1:**
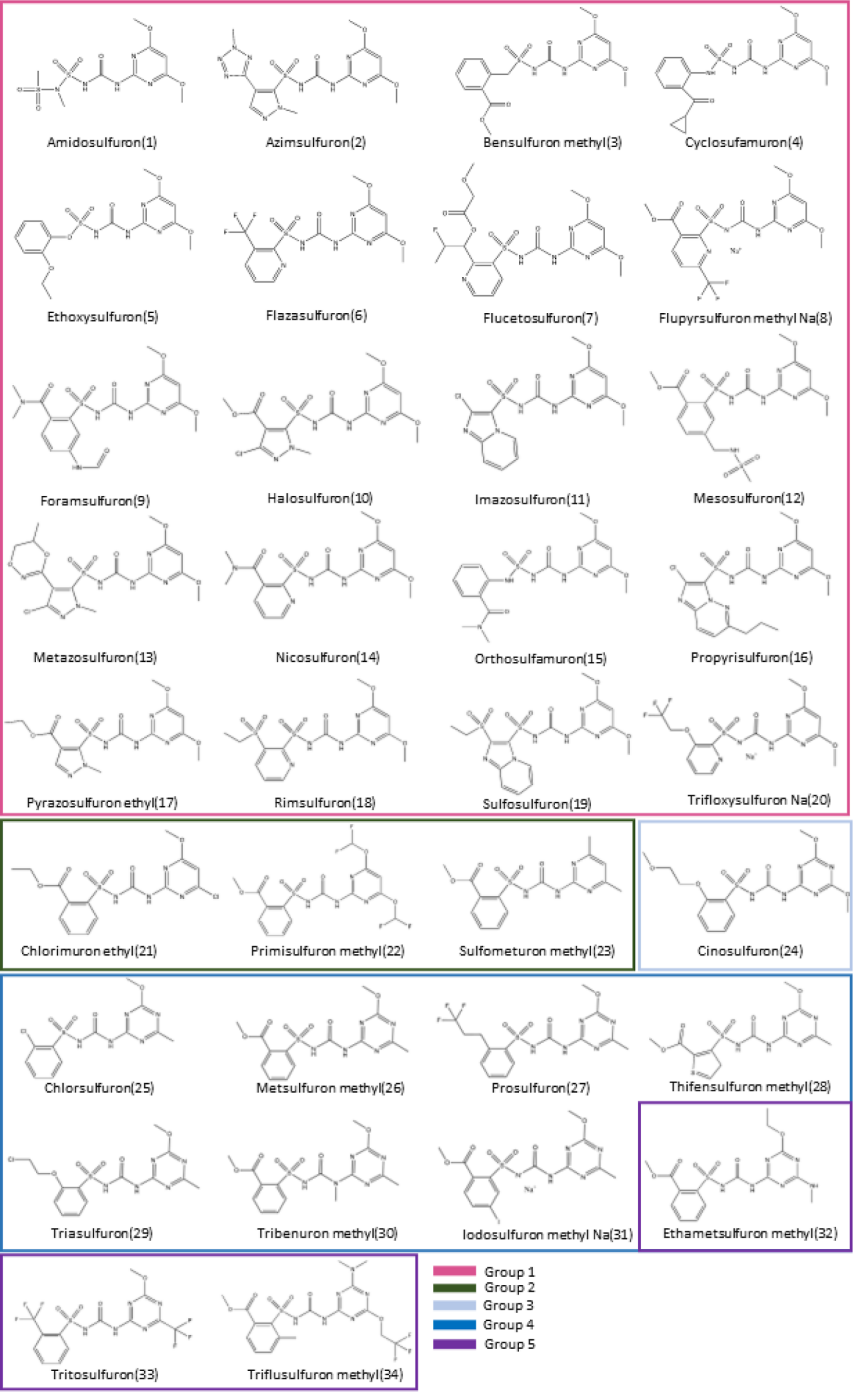
Set of the 34 molecules of studied sulfonylureas.

In Figure 1, we depict the entire collection of 34 sulfonylurea molecules under study. To enhance clarity and depth of analysis, we have further categorized them based on the characteristics of the R1, R2, and R3 groups. This categorization is clearly marked in Figure 1 using distinct color schemes.

- ***Group 1***: In the whole group R1 = R2 = –OCH_3_, with R3 serving as the variable substituent. Except for amidosulfuron, all R3 groups contains an aromatic ring. The X position is occupied by a carbon atom, resulting in a pyrimidine structure.
- ***Group 2***: In this category, the R1 and R2 show variability, R3 is a phenyl ring with an ester in position 2, while X remains a carbon atom.
- ***Group 3***: It mirrors group 1 in many aspects. However, the distinguishing feature is the replacement of the carbon atom at the X position with a nitrogen atom, leading to the formation of a 1,3,5-triazine structure.
- ***Group 4***: In this group, R1 = –OCH_3_ and R2 = –CH_3_, while X is a nitrogen atom, and R3 varies among the molecules.
- ***Group 5***: Here, all R1, R2, and R3 groups display variation, with the X position consistently being a nitrogen atom.

Through this structured approach, we aim to provide a clear and systematic exploration of the sulfonylurea molecules. It must be noted that in three cases, compounds 8, 20, and 31, the HRAC classification indicates the complex of deprotonated form with Na^+^. For the sake of consistency and to describe all sulfonylureas as neutral compounds in those cases, we study the molecule before deprotonation, i.e., keeping intact the urea (−NH–C(=O)–NH−) substructure. It is also important to note that tribenurom methyl (30) is the only compound in this study where in the urea substructure, one H is substituted by a methyl, i.e., leading to a (−NH–C(=O)–N(CH_3_)−) substructure.

## Computational Details

### Tautomer and Conformers

To explore all the potential tautomers and conformers of sulfonylurea herbicides, we have used the CREST software.^15,16^ This software employs semiempirical tight-binding techniques in conjunction with a meta-dynamic driven search algorithm, with an energy threshold set at 30 kcal/mol. This approach yielded a variety of tautomers. Subsequently, to scrutinize the conformation of each tautomer, we employed CREST once more, adjusting the energy threshold to 6 kcal/mol for this phase. This led to the extraction of the structures of several conformers for each possible tautomer. We will refer hereafter to these calculations as “XTb-GFN2 level of theory”.^17^

In our efforts to fine-tune the free energies of the identified conformers, we employed the CENSO program^18^ and conducted calculations at the DFT level. We optimized using the composite PBEh-3c method^19^ and the def2-mSVP basis set, which has been proven to give quite accurate results for the selection of the most stable conformers. The final free energies were then calculated using the wB97x-D3 method^20^ in conjunction with the def2-TZVPP basis set.^21^ All conformers needed to account for 99% of the Boltzmann distribution were selected.

All the calculations were performed in both gas phase and in solution, with the solution phase simulated using the implicit SMD model.^22^ We selected water and octanol as solvents due to their relevance in environmental research. Octanol solvent is used in different physicochemical parameters that determine the environmental behavior of pesticides as the water–octanol partition coefficient. This parameter, defined as the ratio of the equilibrium concentrations of the two-phase system consisting of water and *n*-octanol, mimics partitioning between water and biotic lipids. This parameter is characteristic of the liphophility of the molecule and gives an indication of the compound’s tendency to accumulate in biological membranes and living organism. Its determination gives data required for the registration of new organic chemical. It is considered that substances with a log *K*_ow_ value higher than 3 show a high risk of bioaccumulation in organisms according to European Chemicals Agency (ECHA). Furthermore, the polarity of a molecule is strongly correlated with *K*_ow_. Nonpolar analytes are characterized by log *K*_ow_ values above 4–5, whereas polar analytes have log *K*_ow_ values below 1 or 1.5. Nonpolar pesticides having low solubilities have a high potential to adsorb on soil or aqueous particulate matter in the environment.

We further refined the DFT calculations of the conformers in octanol, water, and the gas phase employing the Gaussian software suite of program.^23^ This entailed an optimization using the B3LYP-D3/6-311+G(d,p) level of theory,^24−26^ incorporating frequency calculations. TD-DFT calculations were also done with the same functional and basis set to derive the first ten excitations and the associated UV–vis spectra. Concluding our process, a single-point calculation was performed at the B3LYP-D3/6-311G+(3df,2p) level of theory. This is the same procedure we are following systematically to explore other families of pesticides; all results for all conformers from the above-mentioned calculations can be visualized and downloaded from the web of the SEPIA project (www.sepia-pesticides.es).

### Topological Analysis

The topological analysis of electron density was conducted using the quantum theory of atoms in molecules (QTAIM)^27,28^ complemented by the non-covalent interactions (NCI) approach^29,30^ and natural bond orbital (NBO) population analysis.^31^ These methodologies are standard tools to discern and categorize intra- and intermolecular interactions within molecular systems. In our study, the descriptors from QTAIM that were harnessed to characterize these interactions were founded upon electron density and energy densities observed at bond critical points (*bcp*). Specifically, we focused on the electron density at the *bcp* and its Laplacian. As per Bader’s theory: (1) elevated values of density, denoted as ρ, signify robust bonds. (2) The Laplacian value, ∇^2^ρ, reveals the nature of the interaction: typically, negative values correspond to localized covalent (open shell) interactions, while positive values relate to noncovalent (closed shell) interactions. For an interaction to be classified as a hydrogen bond (HB), it must meet specific criteria: the ρ should fall within the range of 0.002–0.040 a.u. and the ∇^2^ρ should be between 0.024–0.139 a.u. as established by Koch and Popelier.^32^

The non-covalent interactions (NCI) method serves to detect both inter- and intramolecular interactions by pinpointing regions of minimal electron density between atoms where the reduced density gradient approaches zero. Based on this, interactions can be classified. When electron density is concentrated (λ_2_ < 0), the interaction is deemed “stabilizing”. Conversely, when there’s a depletion of electron density (λ_2_ > 0), it is labeled “destabilizing”.

Regarding the NBO analysis, we have focused on examining the natural charges of atoms and groups of atoms to discern changes among different compounds and to identify parameters that can be incorporated into upcoming QSAR and QSPR studies.

### Property Relationships

One of the goals of the present study is to identify properties obtained from the calculations that can be used as descriptors (input parameters) in subsequent QSAR analysis. In particular, we have explored the following parameters obtained in the DFT calculations:

(a) Properties obtained in the optimization and frequency calculations at B3LYP-D3/6-311+G(d,p) level: total energies (*E*_tot_), Gibbs free energy (*G*_tot_), enthalpy at 298 K (*H*_tot_), entropy (*S*_tot_), constant-volume heat capacity (*C*_*v*_), and zero-point energy correction (ZPE).
(b) Properties obtained from the single-point calculations at B3LYP-D3/6-311+G(3df,2p): energy (*E*_SP_), dipole moment (DM), isotropic polarizability (α_i_), and nuclear repulsion energy (*E*_nuc_). For calculations performed in solvents like water or octanol, we extracted the cavity surface area (CS_a_) and cavity volume.
(c) Using the time-dependent DFT method at B3LYP-D3/6-311+G(d,p), we explored the first 10 excited states. From these calculations, the visible–UV spectra were simulated, using the position and intensities of the transitions. From these calculations, the position (λ_1_) and its intensity (λ_1*i*_) for the first band were evaluated, as well as the same parameters for the second band (λ_2_ and λ_2*i*_).
(d) We analyzed reactivity indices based on conceptual DFT,^33,34^ which also involved comparing properties such as the energy of the HOMO (*E*_HUMO_) and LUMO (*E*_LUMO_), calculating the HOMO–LUMO gap  and derived properties such as hardness (), softness () electronegativity (χ = −μ), and electrophilicity ().

As will be discussed in the next section, we aim also to incorporate other parameters derived from the structure, charge distribution, and results from QTAIM, NBO, and NCI analysis.

The Pearson correlation matrix was employed to uncover relationships between parameters and look for correlations among various physicochemical properties of the studied conformers and the parameters obtained from theoretical calculations.

## Results and Discussions

### Stable Tautomers of Selected Sulfonylurea Compounds

To analyze structural trends in sulfonylureas and prior to a comprehensive conformational analysis, we first conducted a study to identify the stable tautomeric forms. All sulfonylureas have two NH groups and one C=O in their urea part, plus at least two N in the pyrimidine/triazine ring able to accept one H. In some cases, neighbor groups can also contribute to the tautomerism, given potentially a large number of possible tautomers for each molecule. We have explored, using the protocol defined in the computational details, the possible tautomers for five selected sulfonylureas, and to ensure a broad representation of the compounds under examination, we have selected one molecule from each group defined in Figure 1: bensulfuron methyl (3), chlorimuron ethyl (21), cinosulfuron (24), triasulfuron (29), and tritosulfuron (34). We want to stress that for each starting tautomer structure, a full conformational analysis has been carried out using CREST-CENSO and final energies refined by reoptimizing the most stable conformers at the B3LYP-D3/6-311G(d,p) level of theory.

**Figure 2:**
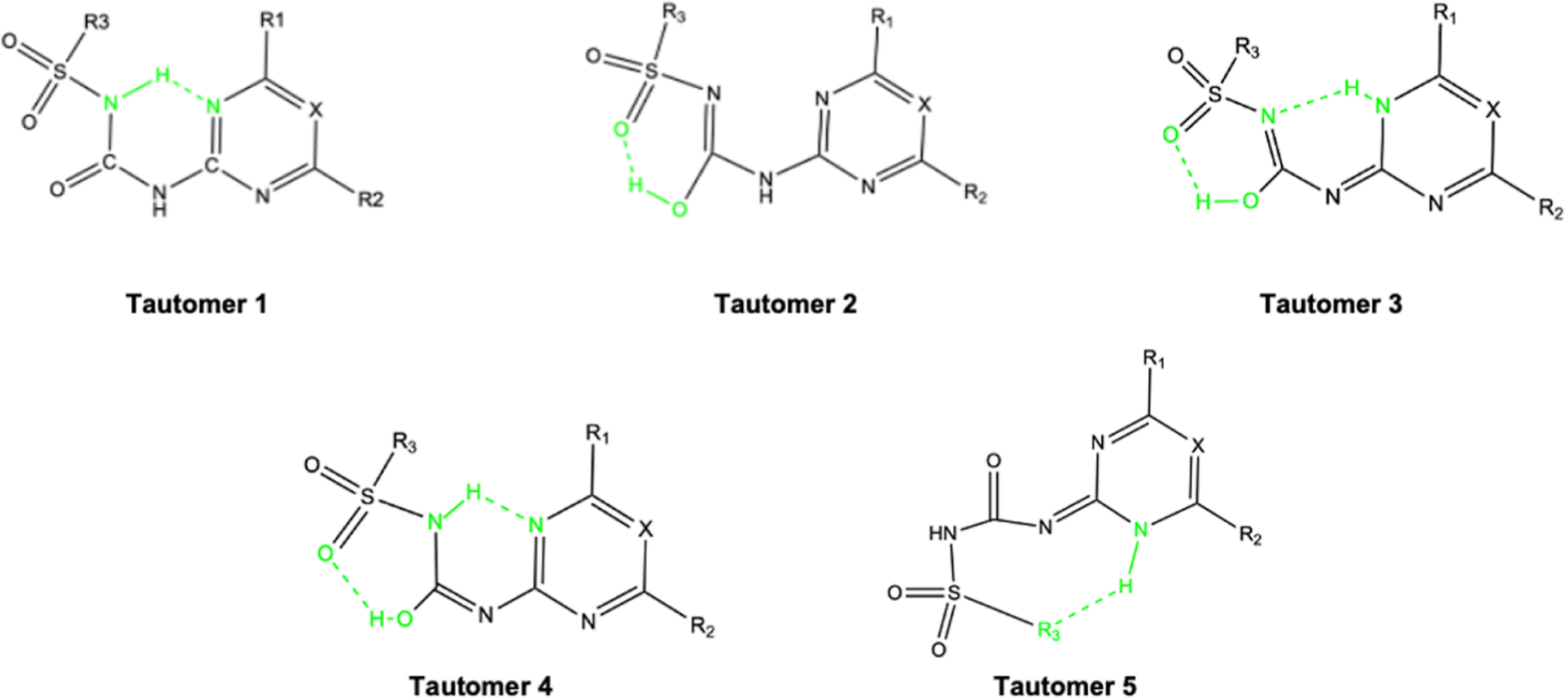
Five tautomer structures for the sulfonylurea pesticides found in a range of 40 kJ/mol at Xtb-GFN2 level. Existing HB in each tautomer are indicated in green.

Figure 2 shows the stable tautomers obtained at XTb-GFN2 level in a range of 40 kJ/mol. Table 1 displays the energy differences among the most stable tautomers for the selected sulfonylurea molecules.

**Table 1:** Relative Stability for Tautomers of the Five Selected Sulfonylureas

molecule	X	R_1_	R_2_	R_3_	Taut	DE (kJ/mol) Xtb-GFN2	DE (kJ/mol) B3LYP-D3/6-311G+(d,p)
bensulfuron methyl	CH	OCH_3_	OCH_3_	*o*-EtOOCAr	**1**	**0.0**	**0.0**
					2	19.9	49.6
					3	34.2	73.2
					5	37.4	82.9
chlorimuron ethyl	CH	OCH_3_	Cl	*o*-EtOOCAr	**1**	**0.0**	**0.0**
					2	29.7	29.3
cinosulfuron	N	OCH_3_	OCH_3_	*o*-CH_3_O(CH_2_)_2_OAr	**1**	**0.0**	**0.0**
					2	27.2	63.6
triasulfuron	N	CH_3_	OCH_3_	*o*-HO(CH_2_)_2_OAr	**1**	**0.0**	**0.0**
					2	25.7	39.5
tritosulfuron	N	OCH_3_	CF_3_	*o*-CF_3_Ar	**1**	**0.0**	**0.0**
					2	21.5	57.5
					4	40.7	73.2

An energy assessment identifies tautomer 1, in which urea group remains as –NH–C(=O)–NH– and an HB is formed between the NH and the pyrimidine/triazine ring (see Figure 2) as the most stable across all examined cases. Tautomer 2 is always the second most stable one. In this case, the urea group tautomerizes to amino/enol –N=C(−OH)–NH– and a HB is formed between the OH and one oxygen of the sulfonyl group. Tautomer 2 is approximately 20–30 kJ/mol less stable than tautomer 1 at the Xtb-GFN2 level. Interestingly, this energy difference becomes larger in all cases (between 30 and 65 kJ/mol) when calculations are performed at a higher level [B3LYP/6-311G+(d,p)], confirming that tautomer 1 is the predominant form in the sulfonylureas. For bensulfuron methyl and tritosulfuron, other tautomeric forms have been found, but they are less stable by more than 70 kJ/mol, and therefore, their role is negligible.

A general conclusion is that the ketone form surrounded by two NH groups (substituted urea) emerges as the predominant configuration among all sulfonylureas. Its high stability among all tautomer seems to be determined by the presence of HBs between two N atoms, with the NH group of the urea part *(N–H)* being the donor and the aromatic N from the pyrimidine/triazine ring the group acting as the acceptor. When doing the subsequent conformational analysis, this HB is always present in the top 5 most stable conformers, indicating that the HB is a determining factor in the stability.

### Structural Characterization of Sulfonylureas

**Figure 3:**
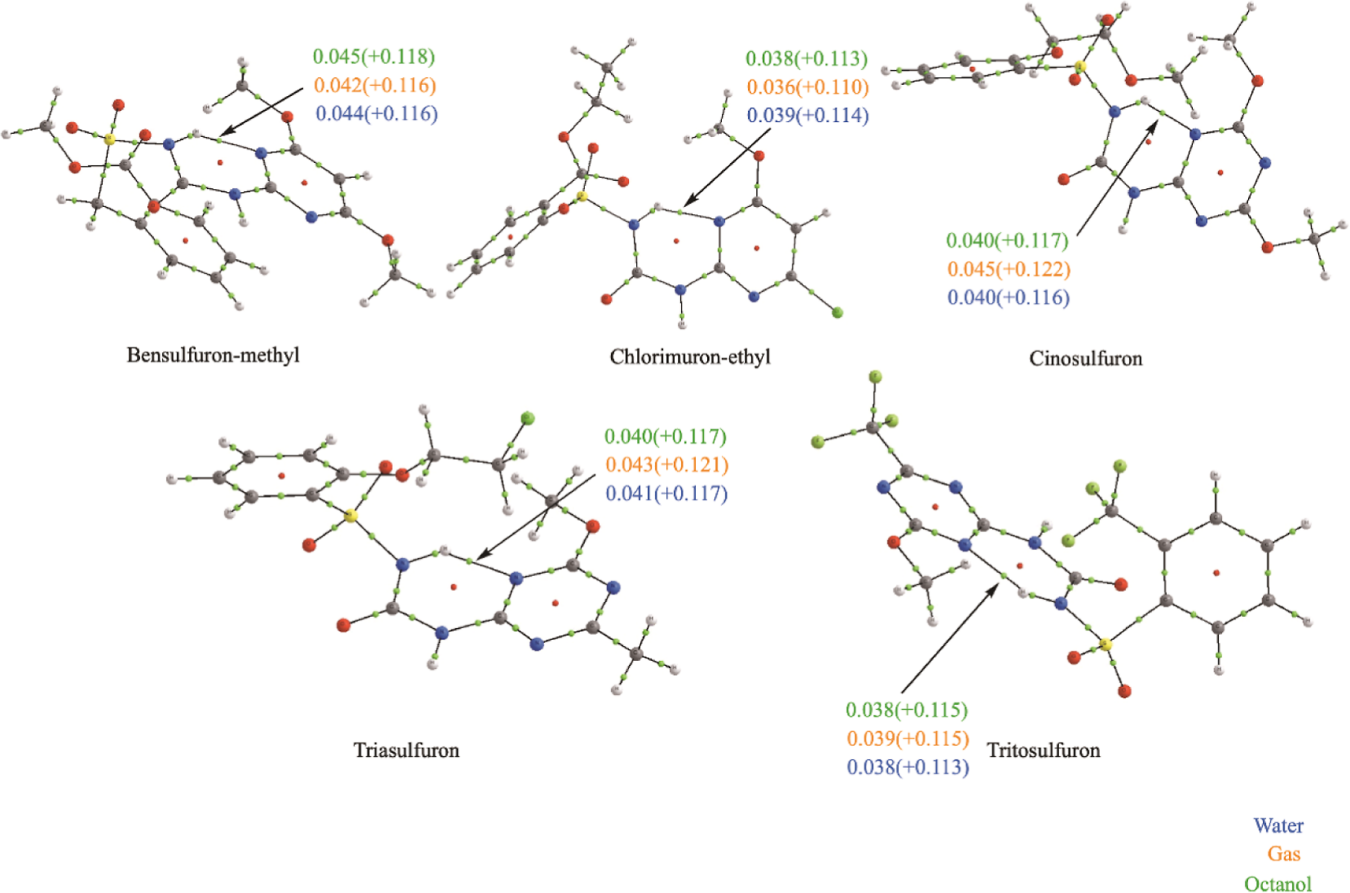
QTAIM graphs of some sulfonylureas. The values of ρ_bcp_ and ∇^2^ρ_bcp_ (in parentheses) of the *NH*···*N* HB considering the different solvents: green for octanol, light brown for the gas phase, and blue for water (values are in a.u.).

In the following discussion, we will refer to the most stable conformation of tautomer 1 in each phase (gas, water or octanol). As stated above, the key factor controlling the structure and stability of sulfonylureas is the presence of a HB. To characterize it along the series, we have done a topological analysis that commenced with examining the density descriptors derived from QTAIM method. In Figure 3, we display, for the HB, the values of electron density ρ_bcp_ and its Laplacian ∇^2^ρ_bcp_ at the bond critical point (bcp), in the three phases (gas, water or octanol). The ρ_bcp_ and ∇^2^ρ_bcp_ values fall within the range of 0.035–0.044 and 0.10–0.12 a.u., respectively, aligning with the typical range for these properties in a HB (0.002–0.040 a.u. for ρ_bcp_ and 0.024–0.139 a.u. for ∇^2^ρ_bcp_) and corresponds to a strong HB between, which is a defining characteristic of the folded primary structure common to all the analyzed sulfonylureas. Notably, the variations in the R1, R2, and R3 groups dictate the three-dimensional structures among the molecules. However, these structural differences always adhere to the framework established by the aforementioned HB. This implies that while substituents may influence the overall molecular configuration, the primary structure is conserved across different sulfonylurea variants.

There are several aspects that can be deduced from the figure:

- There are significant differences in the strength of the HB when comparing different sulfonylureas. For instance, the density at the BCP varies in more than 10% when moving from bensulfuron methyl to chlorimuron methyl.
- In some cases, as cinosulfuron, the differences are also significant when considering water, octanol, or gas phase, but considering the five cases of Figure 3, it is unclear whether HB in water and octanol is stronger than in the gas phase.

These differences are also evident in their HB *NH*···*N* bond lengths: in bensulfuron methyl, it is 1.79 Å in water and in chlorimuron ethyl, it becomes 1.85 Å. These findings underscore the significant variations in HB among sulfonylureas and the influence of the solvent environment.

**Figure 4:**
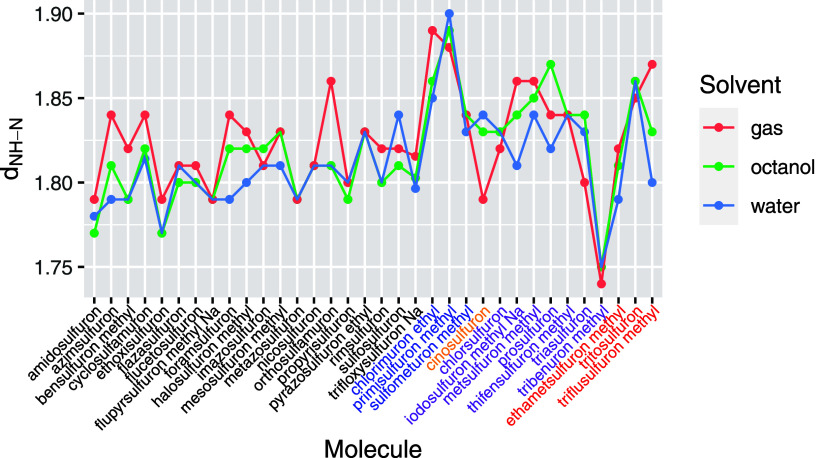
Distance *NH*···*N* (in Å) for all the analyzed sulfonylureas in gas, octanol, and water solvents. Molecules are indicated in the *x*-axis following the same order than in Figure 1. The five groups defined are identified by different colors in the labels of the *x* axis. Values correspond to the most stable conformer for each compound.

To encompass the entire family of sulfonylureas, we present in Figure 4 a graph depicting the variation in NH···N bond lengths across the 34 compounds studied. The hydrogen bonds (HBs) in this series range from moderate to strong, as reflected by the N···H distances, which vary between 1.75 and 1.90 Å. It is important to note that these HN···N distances are sensitive to changes in the solvent environment.

From this figure, some general conclusion can be obtained:

- As a general trend, HBs are stronger in the presence of water, while in gas phase, the HBs are weaker, but there are many exceptions, showing the importance of the influence of R1, R2, and R3 groups on the HB.
- The differences between solvents can be quite high and again reflect changes in R1, R2, and R3 groups. Just to mention two significant cases: for triflusulfuron-methyl (34), where the differences are as large as 0.07 Å, the reason is that for gas and octanol, the R1 group (the one closer to the HB) is –N(CH_3_)_2_ and R2 is –OCH_2_CF_3_, while for water, the 1,3,5-triazine ring is rotated and R1 corresponds to –OCH_2_CF_3._ In the case of orthosulfumaron (15), where large differences are also observed (0.05 Å), the reason is more subtle; in this case, the phenyl ring in R3 changes its 3D orientation, and in the gas phase, it is practically coplanar with the *NH*···*N* bond, while in water and octanol, it is more bended (all structures can be explored and rotated in http://app.sepia-pesticides.es).
- Regarding the differences in the HN strength among sulfonylureas, the larger influence is given from the R1 substituent. The ones with the weaker HBs [chlorimuron ethyl (21) and primisulfuron methyl (22)] correspond to the cases where R1 is either −Cl or –CH(CF_3_)_2_, i.e., they are groups with a large electron-withdrawing effect, decreasing the capacity of N3 to act as a hydrogen acceptor. This also explains why values are relatively constant in the group 1, as in this group, R1 is kept the same (−OCH_3_). The fact that the pyrimidine ring is substituted by the 1,3,5-triazine (groups 3, 4 and 5) seems to have little influence.
- Tribenuron methyl (30) exhibits the strongest hydrogen bond (HB) among all the sulfonylureas, a phenomenon that can be readily explained by the fact that it is the only molecule in this group where one hydrogen in the urea unit has been replaced by a −CH_3_ group. This substitution significantly affects the character of the remaining NH group in the urea, enhancing its effectiveness as a hydrogen-bond donor.

**Figure 5:**
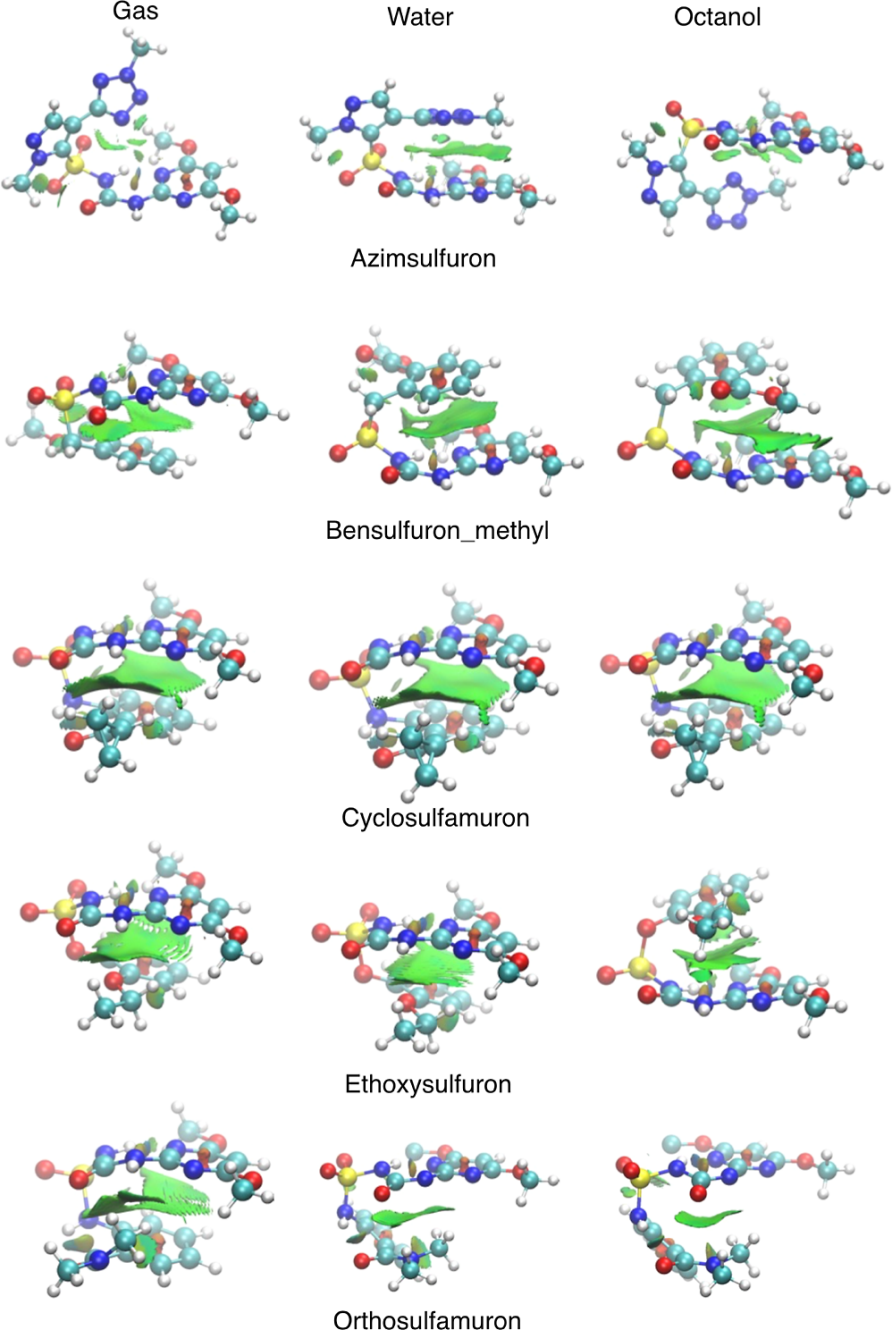
NCI plot of the sulfonylureas showing π–π stacking interactions.

On top of the HB, a second factor that we have identified to explain the overall structure of sulfonylureas is that in certain instances, molecular stability is further augmented by robust π–π interactions. These interactions occur between the diazine/triazine ring and another aromatic ring present in the molecule. Notably, such π–π interactions were detected in five specific cases: azimsulfuron (2), bensulfuron_methyl (3), cyclosulfamuron (4), ethoxisulfuron (5), and orthosulfamuron (15). With the exception of azimsulfuron, these are the only molecules in which an atom with sp^3^ hybridation (−CH_2_–, –NH– or –O−) is bonded to the sulfonyl group. In all other sulfonylureas, the sulfonyl group is directly bonded to an sp^2^ carbon belonging to an aromatic ring. This additional atom provides sufficient flexibility for the entire molecule to facilitate π–π interactions, as illustrated in Figure 5. In azimsulfuron, this flexibility to form π–π stacking arises from the presence of two five-membered rings, though the interaction is less effective. While NH groups in the urea could also confer flexibility, the formation of a hydrogen bond forces the –NH–C(=O)–NH–S– group to align in the same plane as the pyrimidine/triazine ring, thereby preventing the formation of a π–π interaction when R3 is an aromatic ring.

To characterize π–π interactions, we have used the NCI analysis. We observe a pattern of weak intramolecular interactions among the different substituent groups. This is clearly illustrated in Figure 5, which shows a broad area of weak interactions between the two rings, interspersed with small zones exhibiting varying degrees of attractive forces (depicted in green). This pattern is typical of the spatial distribution seen in π–π stacking interactions. Notably, the distribution of these attractive forces is not uniform, suggesting a competition between different molecular forces. This observation confirms the overall weak nature of the π–π stacking interactions, which generally assume a parallel shape. However, exceptions are observed in the case of azimsulfuron (2) in gas and octanol phases and in the case of orthosulfamuron (5) in water and octanol phases, where the interactions appear to be approximately T-shaped (see Figure 5).

The π–π stacking interactions also influence the HB distances, resulting in more consistent *NH*··· *N* distances in water and octanol, thereby stabilizing the molecular structure across different solvents. We also note that the nature of the π–π stacking interaction, whether T-shaped or parallel-shaped, seems to affect the hydrogen bond distances across different phases. For instance, azimsulfuron exhibits different *NH*···*N* distances between octanol and water phases, whereas orthosulfuron shows similar *NH*···*N* distances in these phases. This suggests that the specific type of π–π interaction can influence the hydrogen bond dynamics in various solvent environments.

One structural parameter that can be easily defined to characterize those cases where π–π stacking is present is the sphericity parameter (SP) defined as^35^
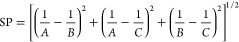
where *A*, *B,* and *C* are the rotational constants (in GHz). This parameter varies from 0 for a perfect sphere up to infinity for an ellipsoid of infinite eccentricity. The advantage of using it is that it can be easily defined for all the molecules and serve also to rapidly identify those cases where competing conformers for the same substance have large differences between their 3D structures, for instance, if π–π stacking appears or changes between conformers and if the overall shape of the molecule changes with the solvent.

**Figure 6:**
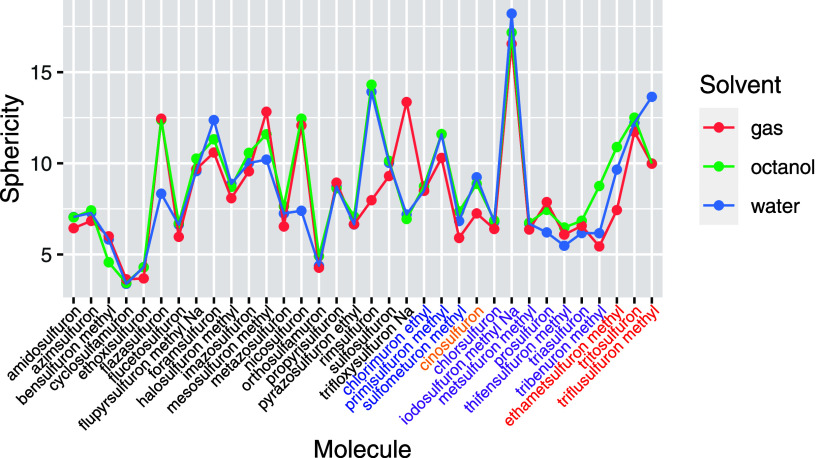
Sphericity parameter for all the analyzed sulfonylureas in gas, octanol, and water solvents. Molecules are indicated in the *x*-axis following the same order as shown in Figure 1. The five groups defined are identified by different colors in the labels of the *x* axis. Values correspond to the most stable conformer for each compound.

**Figure 7:**
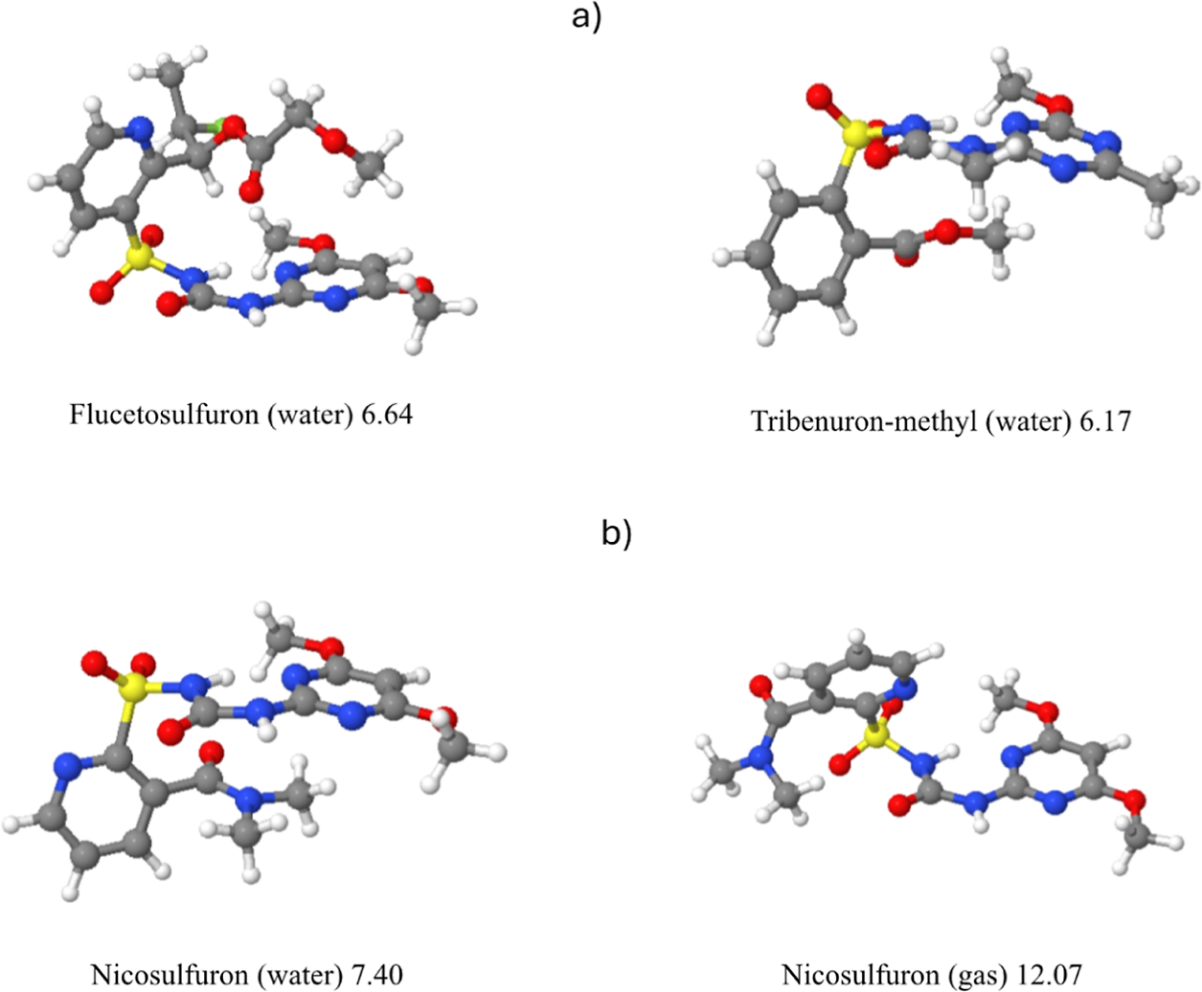
(a) Examples of systems flucetosulfuron (7) and tribenuron-methyl (31) showing small values of SP and not presenting π–π interactions. (b) Structures predicted as the most stable one for nicosulfuron (14) in gas and water.

Figure 6 displays the variation in SP across the entire series of sulfonylureas, illustrating that SP is a highly sensitive parameter to the molecular shape, with values exhibiting a broad range of variation throughout the series. All compounds that exhibit π–π stacking present SP values below 6. Additionally, molecules with large R3 substituents can adopt a “bowl” shape and also show low SP values, as exemplified by flucetosulfuron (7) and tribenuron-methyl (30) (see Figure 7).

In general, values in gas, octanol, and water run in parallel, indicating that the overall molecular structure does not depend on the solvent, but there are noticeable exceptions as nicosulfuron (14). In this case, the different orientation of the R3 group gives the large difference between SP values in gas(octanol) and water, as shown in Figure 7.

In summary, despite the variability in structure and functional groups of these molecules, we could discern certain consistent behaviors, such as the presence of a hydrogen bond, the influence of the solvent, and the occurrence of π–π stacking interactions.

### Electronic Structure and Charge Distribution in Sulfonylureas

**Figure 8:**
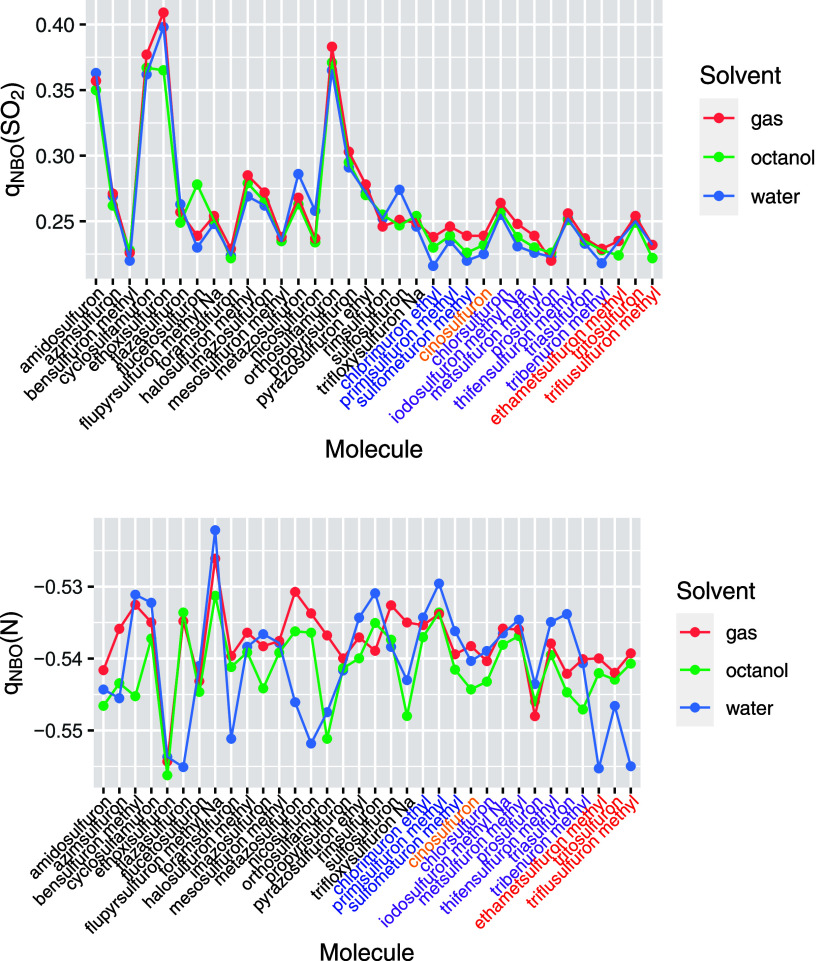
Charge of the –S(=O)_2_– group and the N acting as H donor in the HB for all the analyzed sulfonylureas in gas, octanol, and water solvents. Molecules are indicated in the *x*-axis following the same order as shown in Figure 1. The five groups defined are identified by different colors in the labels of the *x* axis. Values correspond to the most stable conformer for each compound. Charges have been evaluated using the NBO method at B3LYP/6-311+G(d,p) level.

The sulfonyl and urea groups are common elements across the entire family of compounds. To investigate how the properties of these two groups vary among the different compounds, we analyzed the total charge on various atoms within these groups. As mentioned in the introduction, one reason for this analysis is to determine whether these quantities could serve as viable parameters in QSAR models. For these parameters to be useful, they must be sensitive (i.e., they should vary across the series) and not directly correlated with other parameters. Figure 8 illustrates the variation in the total NBO charge on the SO_2_ group and the nitrogen atom that acts as a hydrogen donor in the hydrogen bond.

As depicted in the figure, the charges across all sulfonylureas are relatively constant, with the charge on nitrogen varying by less than 0.03 (a.u.) across the series. The variation is more pronounced for the sulfonyl group (0.15 a.u.), but it primarily depends on the nature of the atom attached to sulfur (R3 group). For instance, the charge becomes depopulated when the attached atom is nitrogen, as seen in amidosulfuron (1), cyclosufamuron (4), or orthosulfamuron (15), and a similar effect occurs when it is oxygen, as in ethoxysulfuron (5). For the remaining compounds, the variation in the charge on sulfur is less than 0.05 a.u. Similarly, the consistent value observed in the nitrogen charge (shown in Figure 8) reflects that this nitrogen is invariably connected to one sulfonyl and one carbonyl group and to a hydrogen forming a similar hydrogen bond (HB). These findings indicate that charges on these groups would likely have little utility as parameters in QSAR models due to their minimal variability.

**Figure 9:**
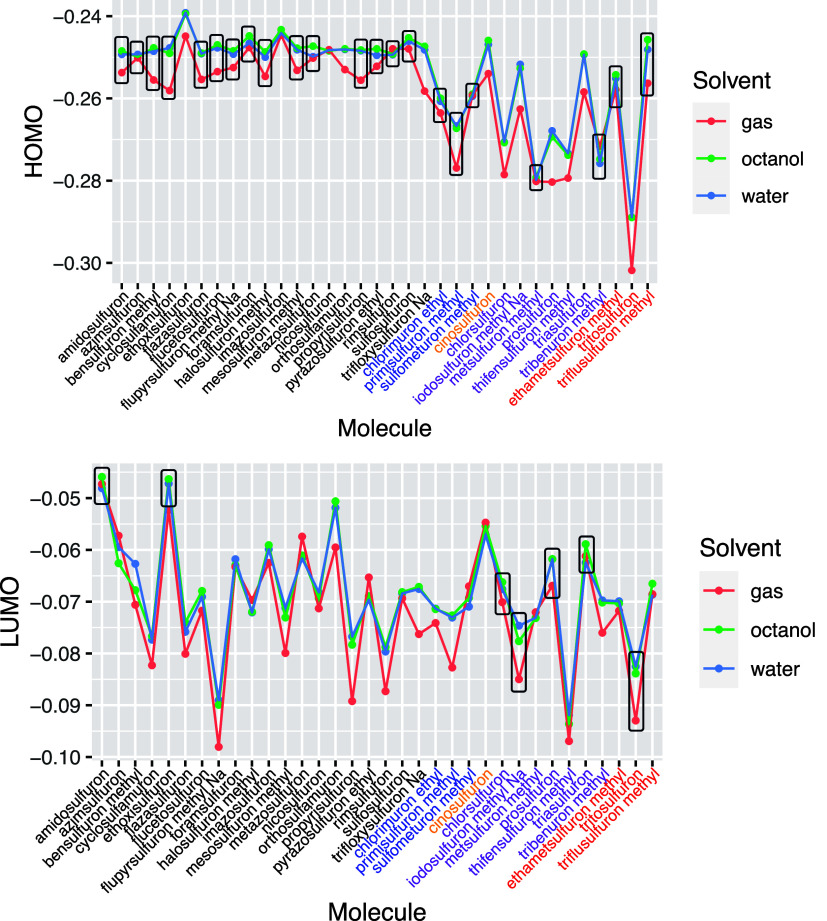
Energies (in a.u.) of the HOMO and LUMO orbitals for all the analyzed sulfonylureas in gas, octanol, and water solvents. Data inside boxes indicate that this orbital is located in the pyrimidine and 1,2,3 triazine rings.

A common parameter used in QSAR models is the energy of the HOMO and LUMO orbitals. Figure 9 analyzes how the energies of these orbitals vary across the series. In group 1, the HOMO is predominantly distributed in the pyrimidine ring, where R1 and R2 are consistently −OCH_3_, resulting in a relatively stable HOMO energy within this group. Conversely, the LUMO is allocated on the aromatic rings associated with R3, exhibiting much greater variability in its energies. For group 2, the HOMO remains in the pyrimidine ring, but variations in R1 and R2 lead to different HOMO energies. In groups 3, 4, and 5, which feature the 1,2,3-ring structure, the distribution of the HOMO is not consistently in this ring, leading to a larger variation in the energies of the frontier orbitals. This variability persists even in group 4, where the R1 and R2 groups remain constant. This analysis illustrates the significant impact that substituent variations can have on the orbital energies, which is crucial for understanding molecular interactions in QSAR models.

### Correlations between Properties

**Figure 10:**
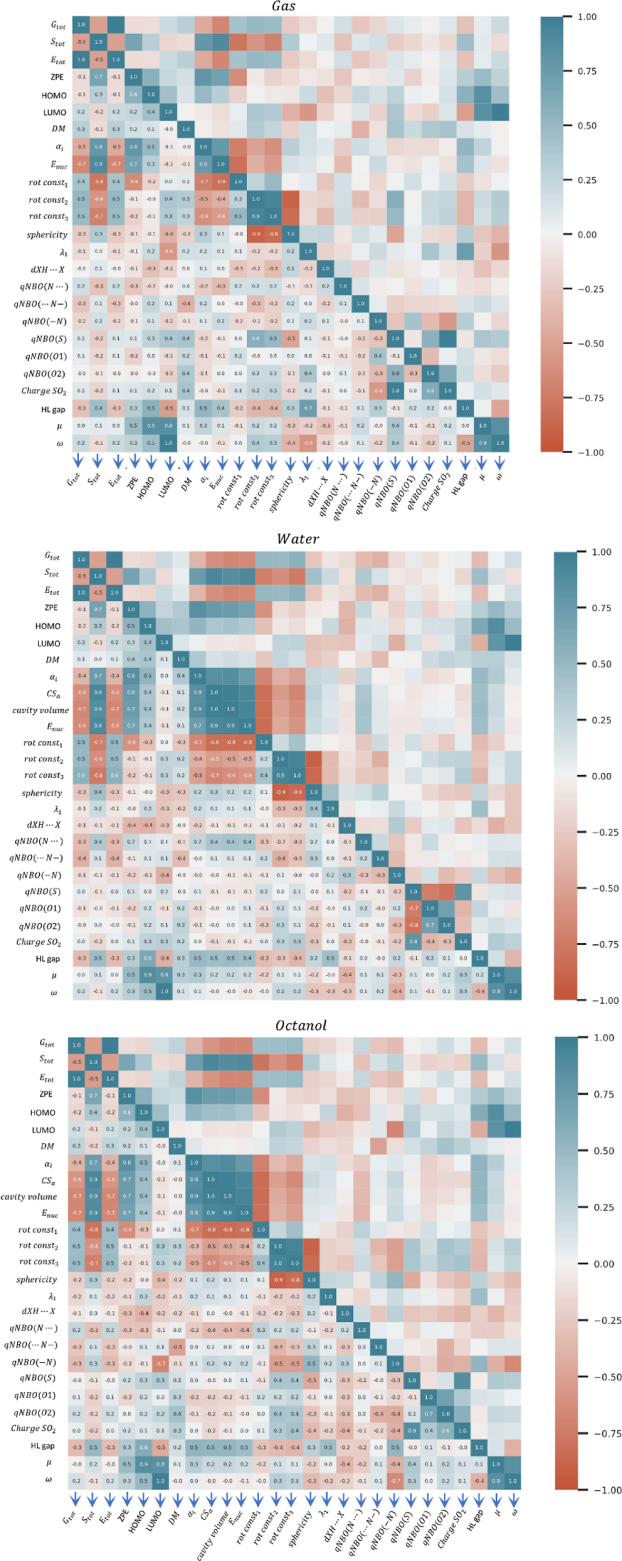
Correlation matrix of theoretical descriptors in the three phases studied.

The objective of our study of the 34 sulfonylureas, as outlined in the introduction, is to identify patterns that could enable us to categorize these substances and predict their physicochemical behaviors. Prior to initiating predictions, it is crucial to compile a comprehensive data set of their properties. For this purpose, we have analyzed many properties derived from DFT calculations performed on the five most stable conformers of each sulfonylurea in each solvent. To uncover potential correlations among these properties, we employed Pearson correlation matrices, which help visualize the strength and direction of relationships between variables. These matrices are color-coded, with a gradient extending from blue to red. Within this spectrum, blue signifies positive correlations, red denotes negative correlations, and the intensity of the color corresponds to the strength of the correlation. The correlation coefficients themselves range from −1, indicating a perfect inverse correlation, to +1, denoting a perfect direct correlation. A value of 0 suggests no correlation. By interpreting these matrices, as exemplified in the accompanying Figure 10, we aim to extract meaningful insights that could inform the classification and prediction of sulfonylurea properties. The properties under scrutiny are the ones listed in the computational details and the structural parameters introduced in the previous section HB distances, SP and charges on the sulfonyl and NH. In our analysis, considering the large number of properties under examination, a correlation is deemed reasonable if the correlation coefficient is 0.5, whether positive or negative.

As expected, there is a strong correlation (correlation values close to 1) among the energy variables such as total energy from optimization (*E*_tot_), enthalpy (*H*_tot_), Gibbs free energy (*G*_tot_), and single-point calculation energy (*E*_SP_). To simplify the figure, only the last two values are kept. Similarly, there is a very high correlation between the entropy (*S*) and the constant volume heat capacity (*C*_*v*_), and only the former is reported.

Polarizability is a property that can be related to the molecular reactivity; in the case of sulfonylureas, a large component of the global polarizability can be attributed to the high polarizability of the sulfur atom. The correlation of polarizability with the energetic properties *G*_tot_ and *E*_SP_ exhibits a correlation coefficient of approximately −0.5. The correlation with *S*_tot_ and ZPE is stronger, presenting coefficients of 0.8. However, when considering solvents such as water and octanol, the strong correlations persist only with *S*_tot_ and ZPE, whereas they diminish with *G*_tot_ and *E*_SP_. Interestingly, in the solvent environment, these same parameters (*S*_tot_ and ZPE) show a pronounced correlation with the solvent’s cavity surface area and volume, indicated by high correlation coefficients. This observation underscores the influence of the solvent environment on the polarizability-related properties of the molecule and its associated reactivity.

To assess the impact of radiation on these compounds, it is essential to analyze the wavelength of their first excitation in the ultraviolet spectrum (λ_1_). This wavelength correlates with the HOMO and LUMO orbitals and, by extension, with various indices derived from conceptual DFT. Analysis of the correlation graph reveals that λ_1_ exhibits an inverse correlation with the LUMO (−0.6) and a positive correlation (0.7) with the HOMO–LUMO gap. The weak correlation with the HOMO reflects that it remains relatively constant across all compounds, as discussed above, and contrasts with the more variable LUMO. This variation explains the correlations observed between λ_1_ and the HOMO–LUMO gap, which subsequently elucidates the relationships with chemical potential, electrophilicity, and LUMO values. In solvent environments such as water and octanol, these correlations do not persist.

Regarding the conceptual DFT parameters, we included only the chemical potential, μ, and electrophilicity, ω, in the plots shown in Figure 10 due to the similar correlations observed with other parameters. Neither parameter shows strong correlations in any solvent, achieving the highest correlations with the zero-point energy (ZPE), λ_1_, and the qNBO(N..) in octanol. The same trends are observed with just the HL gap; however, in solvents like water and octanol, it shows significant correlations with many energetic parameters. The correlations of the HOMO and LUMO energies themselves generally do not match the magnitude of those seen with the HL gap.

With respect to structural parameters and charges, the hydrogen bond (HB) distances and SP exhibit significant variations across the series (see Figures 4 and 6) and show little correlation with the previously discussed parameters, making them strong candidates for inclusion in future QSAR models. Charges of different atoms generally show minimal variation across the series (see Figure 8), with the notable exception being the charge on sulfur. It is also interesting to note the correlation between SP and the charges in the gas phase and octanol, with a coefficient of 0.5. A subtle physical relationship exists among these observations: as discussed earlier, the fact that compounds like bensulfuron_methyl (3), cyclosulfamuron (4), ethoxisulfuron (5), and orthosulfamuron (15) feature an atom with sp^3^ hybridization (−CH_2_–, –NH–, or –O−) attached to sulfur contributes to their 3D flexibility. This attachment also results in a depopulation of charge on sulfur when –NH– or −O– is directly bonded to it.

In summary, to streamline the number of variables in future analyses, we suggest considering the following global parameters: *G*, *S*, λ_1_, α_i_, μ, and the HL gap, together with structural parameters as the SP or HBs distances and the charge on *S*.

## Conclusions

To develop predictive models for various physicochemical and toxicological properties of pesticides, we have studied the sulfonylurea family, classified in group 2 by the Herbicide Resistance Action Committee (HRAC) due to their ability to inhibit the enzyme acetolactate synthase. Initially, we focused on identifying the most probable structures using high-level theoretical techniques. We analyzed different conformers of 34 compounds to assess the most stable ones, aiming to understand their physicochemical properties and the patterns influencing their stability.

We discovered that the stability of these molecules is primarily governed by two types of noncovalent intramolecular bonds: the hydrogen bond involving the NH group adjacent to the sulfonyl group and the π–π interactions between aromatic rings, when present. These bonds were confirmed through population analysis using quantum theory of atoms in molecules (QTAIM) and non-covalent interaction (NCI) techniques. The electron density at critical points, determined by QTAIM, confirms a strong hydrogen bond, while NCI highlights attractive zones between the aromatic groups of some molecules, corroborating the stability provided by π–π stacking.

The strength of the hydrogen bonds across the series of compounds varies little, as the electron density at the critical bonding point does not exceed a 10% variation among the different compounds. These bonds also strengthen in the presence of water, as observed in solvent analysis results. A significant finding is the presence of π–π stacking interactions, which reinforce the hydrogen bonds both in the gas phase and in solvents like water and octanol. Additionally, we have introduced sphericity as a parameter to analyze the shapes of the different molecular structures. This parameter exhibited significant variations across the molecule series and tends to be lower in compounds that feature π–π interactions.

We conducted a statistical analysis of the various physicochemical properties derived from our calculations to assess their potential for inclusion in QSAR predictive models. We have presented the Pearson correlation for 27 properties deduced from high-level theoretical calculations in the three different media. The results reveal some clear correlations as, for instance, the value of energetic parameters and polarizability in the gas phase. In the search of additional parameters to be used in QSAR models, we have identified SP, HB distances, and the charge on S as potential ones to be used in conjunction to others as *G*, *S*, λ_1_, α_i_, μ, and the HL gap.
